# An External Validation Study of the Oakland and Glasgow-Blatchford Scores for Predicting Adverse Outcomes of Acute Lower Gastrointestinal Bleeding in an Asian Population

**DOI:** 10.1155/2021/8674367

**Published:** 2021-01-05

**Authors:** Duc Trong Quach, Uyen Pham-Phuong Vo, Nguyet Thi-My Nguyen, Ly Thi-Kim Le, Minh-Cong Hong Vo, Phat Tan Ho, Tran Ngoc Nguyen, Phuong Kim Bo, Nam Hoai Nguyen, Khanh Truong Vu, Manh Van Dang, Minh Cao Dinh, Thai Quang Nguyen, Xung Van Nguyen, Suong Thi-Ngoc Le, Chi Pham Tran

**Affiliations:** ^1^Department of Internal Medicine, University of Medicine and Pharmacy at Ho Chi Minh City, Vietnam; ^2^Department of Gastroenterology, Gia-Dinh People's Hospital, Vietnam; ^3^Department of Gastroenterology, Cho-Ray Hospital, Vietnam; ^4^Department of Gastroenterology, Can-Tho Central Hospital, Vietnam; ^5^Department of Gastroenterology, Bach-Mai Hospital, Vietnam; ^6^Department of Gastroenterology, Dong-Nai General Hospital, Vietnam; ^7^Department of Gastroenterology, Da-Nang General Hospital, Vietnam; ^8^Department of Gastroenterology, Hue Central Hospital, Vietnam

## Abstract

**Aims:**

This study is aimed at (1) validating the performance of Oakland and Glasgow-Blatchford (GBS) scores and (2) comparing these scores with the SALGIB score in predicting adverse outcomes of acute lower gastrointestinal bleeding (ALGIB) in a Vietnamese population.

**Methods:**

A multicenter cohort study was conducted on ALGIB patients admitted to seven hospitals across Vietnam. The adverse outcomes of ALGIB consisted of blood transfusion; endoscopic, radiologic, or surgical interventions; severe bleeding; and in-hospital death. The Oakland and GBS scores were calculated, and their performance was compared with that of SALGIB, a locally developed prediction score for adverse outcomes of ALGIB in Vietnamese, based on the data at admission. The accuracy of these scores was measured using the area under the receiver operating characteristic curve (AUC) and compared by the chi-squared test.

**Results:**

There were 414 patients with a median age of 60 (48–71). The rates of blood transfusion, hemostatic intervention, severe bleeding, and in-hospital death were 26.8%, 15.2%, 16.4, and 1.4%, respectively. The SALGIB score had comparable performance with the Oakland score (AUC: 0.81 and 0.81, respectively; *p* = 0.631) and outperformed the GBS score (AUC: 0.81 and 0.76, respectively; *p* = 0.002) for predicting the presence of any adverse outcomes of ALGIB. All of the three scores had acceptable and comparable performance for in-hospital death but poor performance for hemostatic intervention. The Oakland score had the best performance for predicting severe bleeding.

**Conclusions:**

The Oakland and SALGIB scores had excellent and comparable performance and outperformed the GBS score for predicting adverse outcomes of ALGIB in Vietnamese.

## 1. Introduction

The prevalence of acute lower gastrointestinal bleeding (ALGIB) which leads to hospitalization has been increasing over the last twenty years [1]. Although there are several risk scores for predicting adverse outcomes of ALGIB, most of these scores fail to accurately predict outcomes when externally validated in other populations [2, 3]. The Oakland score was developed and validated for predicting the absence of adverse outcomes in ALGIB patients in the UK [4]. Although it has been recommended by the British guidelines for stratifying risk of patients presenting with ALGIB [5], there are concerns that the number of patients and hospitals in validation studies is short and the score needs further validation in other populations [6–8]. Besides, the Glasgow-Blatchford score (GBS), which is widely applied in upper gastrointestinal bleeding, has recently demonstrated good performance in patients with ALGIB [3, 9]. To date, there have been no validation studies of the two scores in Asian populations. In Vietnam, we have recently developed and validated a local scoring system, the severe acute lower gastrointestinal (SALGIB) score, to predict severe ALGIB (Table 1) [10]. This score is simpler and easier compared to the two above-mentioned scores and has demonstrated good performance. However, which score has the best performance needs to be investigated. This study is aimed at (1) validating the performance of Oakland and Glasgow-Blatchford (GBS) scores and (2) comparing these scores with the SALGIB score in predicting adverse outcomes of ALGIB in a Vietnamese population.

## 2. Materials and Methods

### 2.1. Setting and Study Design

A multicenter cohort study was conducted at seven secondary and tertiary hospitals across Vietnam (Gia-Dinh People's Hospital, Cho-Ray Hospital, Dong-Nai General Hospital, Can-Tho Central Hospital, Bach-Mai Hospital, Hue Central Hospital, and Da-Nang General Hospital). This study was approved by the Board of Ethics in Biomedical Research of the University of Medicine and Pharmacy at Ho Chi Minh City, Vietnam (numbered 146/DHYD-HDDD, signed on April 21, 2018). The study protocol conforms to the ethical guidelines of the 1975 Declaration of Helsinki. Written informed consent was obtained from all patients or their legal guardians.

### 2.2. Study Population

Patients aged ≥16 years with symptoms suggesting overt ALGIB (i.e., red or maroon colored stools, blood mixed in with the stools, clots per rectum, or the passage of melena without hematemesis) who were admitted and underwent lower gastrointestinal endoscopy at participating hospitals from October 2018 to November 2019 were recruited. Patients who developed ALGIB while admitted for other reasons who had proven findings of upper gastrointestinal bleeding on oesophagogastroduodenoscopy (OGD) were excluded.

### 2.3. Data Collection

Data at admission were recorded in every patient, which included demographic characteristics, symptoms, prior history of ALGIB, comorbidities, vital signs, finding of digital rectal examination, and results of complete blood count, renal function, and coagulation tests.

### 2.4. Treatment

This is an observational study. If patients presented with melena alone or hematochezia and concomitant hemodynamic instability, OGD was performed to exclude upper gastrointestinal bleeding. Lower gastrointestinal endoscopy (sigmoidoscopy or colonoscopy) was performed in all patients to investigate the sources of bleeding, but complete colonoscopy was not a requirement. Patients whose confirmative source of bleeding (i.e., lesions with stigmata of recent bleeding, friable tumors, or colitis) was identified by sigmoidoscopy or those with life-threatening bleeding or comorbidities who could not safely undergo complete colonoscopy were also recruited.

Regarding the hemostatic strategy, gastrointestinal endoscopy was the first choice. If it failed to control bleeding or was not able to identify the source of bleeding, a multidisciplinary team meeting was organized with interventional radiologists and general surgeons to decide the rescue management.

### 2.5. Outcomes

The adverse outcomes of ALGIB in this study consisted of blood transfusion, hemostatic intervention, severe bleeding, and in-hospital death. Blood transfusion was decided according to the recommendations of the National Institute for Health and Care Excellence [11]. Hemostatic intervention was a composite of endoscopic, radiologic, and surgery interventions. Endoscopic interventions were considered as appropriate if endoscopic lesions with stigmata of recent bleeding (i.e., active bleeding, a visible vessel, or an adherent clot) were detected and endoscopically treated. Severe ALGIB was defined as persistent bleeding within the first 24 hours and/or recurrent bleeding after 24 hours of stability accompanied by a further decrease in hematocrit of ≥20% and/or requirement of ≥2 units of packed red blood cells [12].

### 2.6. Statistical Analysis

Categorical data were presented numerically. Quantitative data were tested for normality using the Kolmogorov-Smirnov test, and those with no normal distribution were presented as median and interquartile range (IQR). The Oakland, SALGIB, and GBS scores were calculated basing on the data at admission (Table 1). The performance of each score was measured using the area under the receiver operating characteristic curve (AUC) and was considered as follows: no discrimination (AUC ≤ 0.5), acceptable (AUC: 0.7–0.8), excellent (AUC: 0.8–0.9), and outstanding (AUC > 0.9) [13]. The chi-squared test was used to compare the difference between AUC curves according to the method described by DeLong et al. [14]. All statistical analyses were performed using MedCalc version 19 (MedCalc Software Ltd., Ostend, Belgium).

## 3. Results

### 3.1. Patient Characteristics and Adverse Outcomes

**Figure 1:**
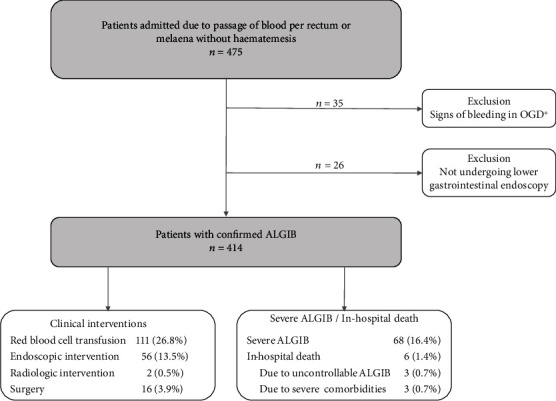
Flowchart of patients recruited for this study. OGD: oesophagogastroduodenoscopy; ALGIB: acute lower gastrointestinal bleeding. ^∗^Upper gastrointestinal bleeding to be excluded by OGD in patients who presented with (i) melena only or (ii) hematochezia and concomitant hemodynamic instability.

The demographic and clinical characteristics of patients in this study are presented in Table 2. The causes of ALGIB in our study are presented in Table 3. And the adverse outcomes of ALGIB are summarized in Figure 1.

### 3.2. Performance of Scoring Systems in Predicting Adverse Outcomes of ALGIB

For predicting the presence of any adverse outcomes, the SALGIB score had comparable performance with the Oakland score (AUC: 0.81 and 0.81, respectively; *p* = 0.631) and outperformed the GBS score (AUC: 0.81 and 0.76, respectively; *p* = 0.002) (Table 4). All scores had acceptable and comparable performance for in-hospital death but no discrimination for hemostatic intervention. For predicting the need of blood transfusion, the SALGIB score also had comparable performance with the Oakland score (AUC: 0.91 and 0.93, respectively; *p* = 0.163) and outperformed the GBS score (AUC: 0.91 and 0.87, respectively; *p* = 0.016). The Oakland score was the best one for predicting severe ALGIB with an AUC of 0.90.

The cut-off points of the Oakland score at ≤14, the SALGIB score at 0, and the GBS score at ≤2 had a sensitivity of about 85% for predicting an uneventful course (i.e., ALGIB without any in-hospital adverse outcomes) in one-third of ALGIB patients (Table 5). And the cut-off points of the Oakland score at 0 and GBS at 0 had the sensitivities of 96.9% and 92.4% for predicting an uneventful course of ALGIB in 10.4% and 17.5% of patients, respectively.

## 4. Discussion

The prevalence of severe ALGIB and in-hospital death in our study was comparable with that reported by Oakland et al. in a multicenter cohort study in the UK [4], but it was lower than that reported in many single-center cohorts [2, 3, 12, 15]. This difference could be partly explained by the fact that the participants in these single-center studies were recruited from tertiary hospitals, and only those performed with colonoscopy were included [3, 15]. As we aimed to evaluate the performance of prediction scores in real-life practice, ALGIB patients in our study were recruited from hospitals with different levels of care across Vietnam, and those who did not have complete colonoscopy were also included.

To the best of our knowledge, our study was the first validation study of the Oakland score in an Asian population. Previously, this score has been validated in the UK and showed good performance for predicting blood transfusion, rebleeding, and the presence of any adverse outcomes [4]. Another cohort study in the US also found that it was the best score for predicting severe ALGIB compared to the GBS, AIMS65, and Strate scores [3]. Our study showed that the SALGIB and the Oakland scores had excellent and comparable performance for predicting the presence of any adverse outcomes in Vietnamese. However, as the former has much fewer components, it would be more convenient for daily practice than the later score. In our study, although the GBS score was outperformed by the two other scores, it still had acceptable discrimination for predicting the presence of any adverse ALGIB outcomes. The GBS score has been well validated for use in upper gastrointestinal bleeding, but some recent studies also reported that it also performed well in patients with ALGIB [3, 9, 16]. In real-life practice, it is sometimes very difficult to locate the source of gastrointestinal bleeding at admission. Our study, therefore, suggested that the GBS score could be considered an alternative prediction tool in such challenging situations.

Regarding the cut-off point for predicting the absence of adverse ALGIB outcomes, an Oakland score ≤ 10 in our study or ≤8 in the previous cohort in the UK had a sensitivity of 96.9% and 95%, respectively [4]. These cut-offs, while having the merit of selecting patients with potentially safe discharge, are not clinically important as only less than one-tenth of patients in both above-mentioned cohorts had such points. In our study, we found that an Oakland score ≤ 14 or a SALGIB score at 0 or a GBS score ≤ 2 helped to predict an uneventful course of ALGIB with a sensitivity of about 85% in one-third of ALGIB patients. These cut-offs, therefore, could be more practical to stratify ALGIB patients for an appropriate level of care.

Our study has some limitations. First, there was no follow-up after hospital discharge in this study. Therefore, it only demonstrated the performance of the scoring systems in predicting in-hospital adverse outcomes. Identifying patients with low risk who are suitable to manage as outpatients needs to be further evaluated in a future study. Second, as only two-thirds of patients in this study underwent colonoscopy, the source of bleeding might have been missed in some patients. Third, the hemostatic strategies at participating sites were divergent depending on the local resources and expertise and could potentially affect the adverse outcomes of ALGIB in this study.

In conclusion, we found that the Oakland and SALGIB scores had excellent and comparable performance and outperformed the GBS score for predicting adverse outcomes of ALGIB in Vietnamese.

## Figures and Tables

**Table 1:** Components of the Oakland, SALGIB, and Blatchford scores.

Oakland score		SALGIB score		Blatchford score	
Age (years)		Heart rate (bpm)		Heart rate (bpm)	
<40	0	<100	0	<100	0
40–69	1	≥100	1	≥100	1
≥70	2	SBP (mmHg)		SBP (mmHg)	
Sex		≥100	0	≥110	0
Female	0	<100	3	100–109	1
Male	1	Hematocrit (%)		90–99	2
Previous ALGIB admission		≥35	0	<90	3
No	0	30–34.9	1	Blood urea nitrogen (mg/dL)	
Yes	1	25–29.9	3	<19	0
DRE findings		<25	5	≥19 and <22.4	2
No blood	0	Platelet count (10^3^/*μ*L)		≥22.4 and <28	3
Blood	1	>150	0	≥28 and <70	4
Heart rate (bpm)		≤150	1	≥70	6
<70	0			Hemoglobin (male) (g/L)	
70–89	1			≥130	0
90–109	2			≥120 and <130	1
≥110	3			≥100 and <120	3
SBP (mmHg)				<100	6
<90	5			Hemoglobin (female) (g/L)	
90–119	4			≥120	0
120–129	3			≥100 and <120	1
130–159	2			<100	6
≥160	0			Chronic disease	
Hemoglobin (g/L)				Hepatic disease	2
<70	22			Cardiac failure	2
70–89	17			Melena	
90–109	13			No	0
110–129	8			Yes	1
130–159	4			Syncope	
≥160	0			No	0
				Yes	2

ALGIB: acute lower gastrointestinal bleeding; DRE: digital rectal examination; SBP: systolic blood pressure.

**Table 2:** Demographic and clinical characteristics of patients in the study.

Characteristics	
Age, median (interquartile range)	60 (48-71)
Sex	
Male, *n* (%)	202 (48.8)
Female, *n* (%)	212 (51.2)
Participating site	
Gia-Dinh People's Hospital, *n* (%)	74 (17.9)
Cho-Ray Hospital, *n* (%)	111 (26.8)
Dong-Nai General Hospital, *n* (%)	43 (10.4)
Can-Tho Central Hospital, *n* (%)	55 (13.4)
Bạch-Mai Hospital, *n* (%)	51 (12.3)
Hue Central Hospital, *n* (%)	40 (9.6)
Da-Nang General Hospital, *n* (%)	40 (9.6)
Previous admission with ALGIB, *n* (%)	115 (27.8)
Comorbidities	
Congestive heart failure, *n* (%)	12 (2.9)
Ischemic heart disease, *n* (%)	42 (10.1)
Chronic obstructive pulmonary disease, *n* (%)	6 (1.4)
Chronic liver disease, *n* (%)	37 (8.9)
Chronic kidney disease, *n* (%)	26 (6.3)
Stroke or transient ischemic attack, *n* (%)	15 (3.6)
Cancer, *n* (%)	38 (9.2)
Hypertension, *n* (%)	147 (35.5)
Diabetes, *n* (%)	45 (10.9)
Preadmission medications	
Aspirin, *n* (%)	20 (4.8)
Clopidogrel, *n* (%)	22 (5.3)
Dual antiplatelet, *n* (%)	8 (1.9)
Warfarin, *n* (%)	2 (0.5)
NOAC, *n* (%)	5 (1.2)
Corticosteroid, *n* (%)	9 (2.2)
NSAIDs, *n* (%)	30 (7.2)
Presenting signs and symptoms	
Heart rate (beat per minute)	86 (80-92)
Systolic blood pressure (mmHg)	120 (110-130)
Melena, *n* (%)	64 (15.4)
Syncope, *n* (%)	2 (0.5)
Blood on DRE, *n* (%)	260 (62.8)
Laboratory data at admission	
Hematocrit (%)	31.8 (24.5-38.4)
Hemoglobin (g/L)	103 (76-126)
Platelet (×10^3^/*μ*L)	242 (191-311)
INR	1.06 (1.00-1.15)
Blood urea nitrogen (mg/dL)	14.2 (11-21)
Creatinine (*μ*mol/L)	0.93 (0.79-1.14)
Types of lower gastrointestinal endoscopy	
Colonoscopy, *n* (%)	264 (63.8)
Sigmoidoscopy, *n* (%)	78 (18.8)
Rectoscopy, *n* (%)	68 (16.4)
Enteroscopy, *n* (%)	5 (1.2)
Abdominal CT scan, *n* (%)	80 (19.3)

Data are *n* (%) or median (interquartile range). NOAC: novel oral anticoagulant; NSAIDs: nonsteroidal anti-inflammatory; DRE: digital rectal examination.

**Table 3:** Sources of lower gastrointestinal bleeding.

Source of bleeding	*n* (%)
Hemorrhoids	120 (29.0)
Inflammatory bowel disease	46 (11.0)
Colon adenocarcinoma	31 (7.5)
Colorectal polyps	31 (7.5)
Diverticulosis	25 (6.0)
Angioectasia	25 (6.0)
Colon ulcers	25 (6.0)
Colitis	23 (5.6)
Benign anorectal disorder (except hemorrhoids)	15 (3.6)
Small intestinal ulcers	7 (1.7)
Small bowel tumor	2 (0.5)
Duodenal pseudoaneurysm rupture	1 (0.2)
Postpolypectomy/posthemorrhoidectomy	11 (2.7)
Unknown	52 (12.6)

**Table 4:** Performance of the Oakland and Glasgow-Blatchford scores in comparison with SALGIB scores in the prediction of adverse outcomes.

	Red blood cell transfusion	Hemostatic intervention	Severe ALGIB	In-hospital death	Any adverse outcome^∗^
	*n* = 111 (26.8%)	*n* = 63 (15.2%)	*n* = 68 (16.4%)	*n* = 6 (1.4%)	*n* = 161 (38.9%)
SALGIB	0.91 (0.88-0.94)	0.53 (0.48-0.58)	0.87 (0.83-0.90)	0.82 (0.78-0.86)	0.81 (0.77-0.84)
Oakland	0.93 (0.90-0.95)	0.52 (0.47-0.56)	0.90 (0.87-0.93)	0.77 (0.72-0.81)	0.81 (0.77-0.85)
	*p* = 0.163	*p* = 0.437	*p* = 0.038	*p* = 0.291	*p* = 0.631
Blatchford	0.87 (0.84-0.90)	0.50 (0.45-0.55)	0.83 (0.79-0.87)	0.76 (0.72-0.80)	0.76 (0.72-0.80)
	*p* = 0.016	*p* = 0.734	*p* = 0.052	*p* = 0.539	*p* = 0.002

Data are presented as areas under the receiver operating characteristic curve and 95% confidence intervals. ^∗^Combined outcome of red blood cell transfusion, hemostatic interventions, severe bleeding, and in-hospital death; *p* values are from the DeLong et al. test.

**Table 5:** Cut-off points of scoring systems for predicting the presence of any adverse outcome.

Oakland score				SALGIB score				Blatchford score			
Score	Sens	Spec	Cum%^∗^	Score	Sens	Spec	Cum%^∗^	Score	Sens	Spec	Cum%^∗^
>5	100.00	0.40	0.2	>0	85.71	45.85	33.6	>0	92.45	23.72	17.5
>6	100.00	1.58	1.0	>1	81.37	70.36	50.2	>1	88.68	43.08	30.8
>7	100.00	2.37	1.4	>2	77.02	74.31	54.3	>2	85.53	48.62	35.4
>8	99.38	5.93	3.9	>3^∗∗^	65.84	87.75	66.9	>3	83.02	54.94	40.3
>9	99.38	7.51	4.8	>4	60.25	92.49	72.0	>4	79.25	60.87	45.4
>10	96.89	15.02	10.4	>5	30.43	94.07	84.5	>5^∗∗^	78.62	65.61	48.5
>11	93.79	23.72	16.9	>6	13.66	99.21	94.2	>6	59.75	78.26	63.6
>12	90.06	30.04	22.2	>7	9.94	100.00	96.1	>7	45.28	86.17	74.0
>13	89.44	37.15	26.8	>8	8.70	100.00	96.6	>8	38.99	90.12	78.9
>14	86.34	46.25	33.6	>9	4.35	100.00	98.3	>9	26.42	94.07	86.2
>15	84.47	51.38	37.4	>10	0.00	100.00	100.0	>10	22.01	96.84	89.6
>16	83.23	58.50	42.3					>11	15.09	98.42	93.2
>17	82.61	63.64	45.7					>12	11.32	99.21	95.1
>18	81.37	66.80	48.1					>13	5.66	100.00	97.8
>19	78.88	69.17	50.5					>14	2.52	100.00	99.0
>20	73.91	73.12	54.8					>15	1.89	100.00	99.3
>21^∗∗^	68.94	82.21	62.3					>16	0.00	100.00	100.0
>22	66.46	83.79	64.3								
>23	60.87	87.35	68.6								
>24	56.52	92.49	73.4								
>25	49.69	94.47	77.3								
>26	43.48	96.44	80.9								
>27	40.99	97.23	82.4								
>28	32.30	98.42	86.5								
>29	24.22	98.42	89.6								
>30	14.29	99.60	94.2								
>31	6.83	99.60	97.1								
>32	3.73	99.60	98.3								
>33	0.00	100.00	100.0								

^∗^Cum%: percentage of the total frequency of patient distribution according to score. Sens: sensitivity; spec: specificity. ^∗∗^Cut-off points determined by the Youden index.

## Data Availability

The data file (.sav) used to support the findings of this study are available from the corresponding author upon request.

## Supplementary Materials

Supplementary Materials The supplementary file includes the TRIPOD Checklist: Prediction Model Development.
